# Exosomes Secreted by Adipose-Derived Stem Cells Following FK506 Stimulation Reduce Autophagy of Macrophages in Spine after Nerve Crush Injury

**DOI:** 10.3390/ijms22179628

**Published:** 2021-09-06

**Authors:** Pao-Jen Kuo, Cheng-Shyuan Rau, Shao-Chun Wu, Chia-Wei Lin, Lien-Hung Huang, Tsu-Hsiang Lu, Yi-Chan Wu, Chia-Jung Wu, Chia-Wen Tsai, Ching-Hua Hsieh

**Affiliations:** 1 Department of Plastic Surgery, Kaohsiung Chang Gung Memorial Hospital and Chang Gung University College of Medicine, Kaohsiung 83301, Taiwan; bow110470@gmail.com (P.-J.K.); sallylin1201@gmail.com (C.-W.L.); rabbit670326@yahoo.com.tw (T.-H.L.); janewu0922@gmail.com (Y.-C.W.); alice8818@yahoo.com.tw (C.-J.W.); flying011401@gmail.com (C.-W.T.); 2 Department of Neurosurgery, Kaohsiung Chang Gung Memorial Hospital and Chang Gung University College of Medicine, Kaohsiung 83301, Taiwan; ersh2127@adm.cgmh.org.tw (C.-S.R.); ahonbob@gmail.com (L.-H.H.); 3 Department of Anesthesiology, Kaohsiung Chang Gung Memorial Hospital and Chang Gung University College of Medicine, Kaohsiung 83301, Taiwan; shaochunwu@gmail.com; 4 Center for Vascularized Composite Allotransplantation, Chang Gung Memorial Hospital, LinKou 33333, Taiwan

**Keywords:** autophagy, sciatic nerve crush injury, exosome, adipose-derived stem cells (ADSC), tacrolimus (FK506), proteomic analysis, isobaric tags for relative and absolute quantitation (iTRAQ)

## Abstract

Macrophages emerge in the milieu around innervated neurons after nerve injuries. Following nerve injury, autophagy is induced in macrophages and affects the regulation of inflammatory responses. It is closely linked to neuroinflammation, while the immunosuppressive drug tacrolimus (FK506) enhances nerve regeneration following nerve crush injury and nerve allotransplantation with additional neuroprotective and neurotrophic functions. The combined use of FK506 and adipose-derived stem cells (ADSCs) was employed in cell therapy for organ transplantation and vascularized composite allotransplantation. This study aimed to investigate the topical application of exosomes secreted by ADSCs following FK506 treatment (ADSC-F-exo) to the injured nerve in a mouse model of sciatic nerve crush injury. Furthermore, isobaric tags for relative and absolute quantitation (iTRAQ) were used to profile the potential exosomal proteins involved in autophagy. Immunohistochemical analysis revealed that nerve crush injuries significantly induced autophagy in the dorsal root ganglia and dorsal horn of the spinal segments. Locally applied ADSC-F-exo significantly reduced autophagy of macrophages in the spinal segments after nerve crush injury. Proteomic analysis showed that of the 22 abundant exosomal proteins detected in ADSC-F-exo, heat shock protein family A member 8 (HSPA8) and eukaryotic translation elongation factor 1 alpha 1 (EEF1A1) are involved in exosome-mediated autophagy reduction.

## 1. Introduction

A remote cell body response in axotomized neurons is induced following a sciatic nerve injury [1]. This strong response indicates the neuronal release of cytokines and chemokines, the induction of neuron-intrinsic growth programs, the activation of resident macrophages [2,3,4], and invasion of macrophages from the peripheral circulation [5,6]. The proliferation of resident and invasive macrophages is abundant and peaks three days after injury [7], with an increased (proportional) influx of hematogenous macrophages after day 4 [7]. The secreted molecules and phagocytosis activity of these macrophages can magnify immune and inflammatory responses in the local milieu, influence the surrounding neurons [8], and further contribute to secondary damage and the further loss of neurons [9].

Autophagy is known to specifically remove and recycle damaged cellular organelles and aggregated proteins to maintain cellular homeostasis [10]. Cells that undergo autophagy exhibit the expression of autophagy markers, such as the microtubule-associated protein 1A/1B-light chain 3 (LC3), the autophagy receptor sequestosome 1 (p62), and lysosomal associated membrane protein 1 (LAMP-1) [11]. For instance, after an optic nerve injury, autophagy is associated with the death of retinal ganglion cells [12,13]. Within a few hours following the optic nerve injury, the optic nerve showed a rapid increase in autophagic vesicles, which spread back to the retinal ganglion cells with LC3 levels being increased as early as 24 h after the injury [14]. Autophagy-related genes such as autophagy-related 5 and autophagy-related 7 are increased in retinal ganglion cells between 3 and 10 days after injury [15]. In addition, insufficient autophagic flux and parallel upregulation of p62 lead to a substantial increase in p62 levels in the optic nerve [16,17]. High levels of autophagy lead to the death of retinal ganglion cells in vivo [14]. Autophagy activation in macrophages of the nervous system is closely linked to neuroinflammation [18,19]. During injury to the central nervous system, autophagy contributes to the regulation of inflammatory responses in resident and invaded macrophages [9]. During injury to the peripheral nervous system, such as spinal nerve ligation, autophagy is also initiated in the macrophages of the spinal segment [20] and participates in the regulation of inflammasome activation and the process of neuropathic pain [18].

Cell-based therapy is currently considered promising for the treatment of peripheral nerve injury [21,22]. Several studies have demonstrated that adipose-derived stem cells (ADSCs) [23,24,25] and even their conditioned medium can promote peripheral nerve regeneration [26,27]. The exosomes might be responsible for the effect of the secretome of the medium by transporting proteins, nucleic acids, and lipids into target cells for intercellular communication [28,29]. It has been reported that exosomes secreted by ADSCs (ADSC-exo) enhance nerve regeneration by stimulating Schwann cell proliferation [30], increasing remyelination [31], and reducing neuronal death [32]. Although the pathogenesis of autophagy in nerve injury is still unclear, autophagy has been proposed as a potential therapeutic target for suppressing cell and tissue injury [18].

The calcineurin inhibitor FK506 was approved by the Food and Drug Administration of United States in 1997 to be used in kidney transplantation to prevent acute rejection [33,34]. FK506 binds to FK506-binding proteins, inhibits the calcineurin/the nuclear factor of activated T cells pathway (NFAT) pathways, and thus suppresses IL-2 gene transcription in T lymphocytes, which is required for T-cell proliferation [33,34]. In addition to the immunosuppressive effect, FK506 had been found to improve nerve regeneration following a nerve crush injury and/or nerve allotransplantation in clinical settings [35,36] and exhibits additional neuroprotective and neurotrophic activities [37]. The combined use of FK506 and ADSCs has been used in cell therapy for organ transplantation [38] or vascularized composite allotransplantation [39,40]. In a mouse model of sciatic nerve crush injury, we showed that the topically sprayed exosomes, which were secreted by ADSCs under the condition of FK506 stimulation (ADSC-F-exo), exhibit similar effects to those of ADSC-exo on enhancing nerve regeneration [41]. Under the hypothesis that the topically sprayed ADSC-F-exo around the crush nerve segment can also rescue the autophagy of macrophages in the milieu surrounding the innervated neurons, this study was performed with the aim to assess the effect of ADSC-F-exo treatment on the autophagy of macrophages in the spinal segments following nerve crush injury, with the additional goal of exploring the potential effective proteins inside the exosomes. In this study, *CSF-1R–GFP*^+^ macrophage Fas-induced apoptosis (MaFIA) transgenic mice, in which macrophages express enhanced green fluorescent protein (eGFP) were used; this facilitated the detection of macrophages in the DRG and spinal segments. Furthermore, isobaric tags for relative and absolute quantitation (iTRAQ) of exosomal protein content were performed to determine potential proteins that can be involved in the autophagy pathway.

## 2. Results

### 2.1. Characterization of Isolated Exosomes

**Figure 1:**
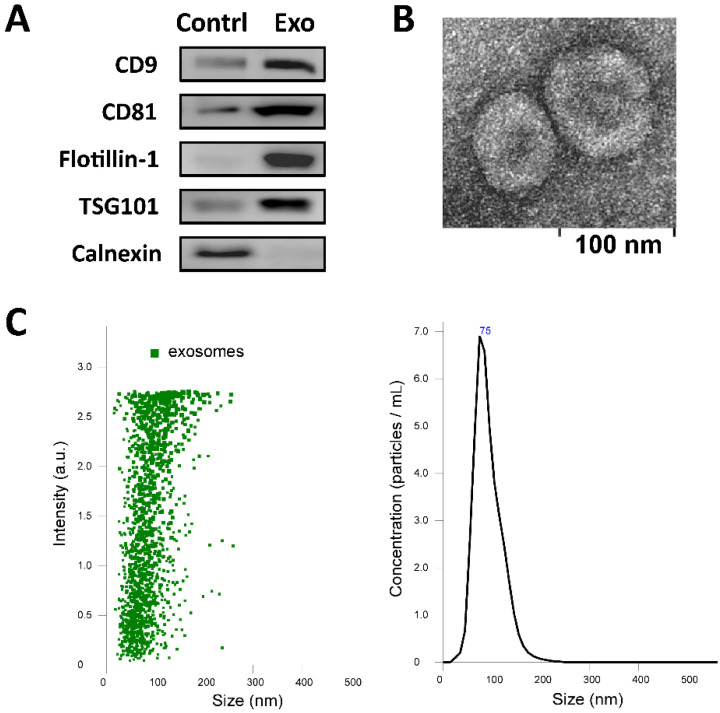
Characterization of isolated exosomes using (**A**) Western blotting for exosomal surface markers of the isolated exosomes (Exo) with cell lysate as the control (Contrl), (**B**) transmission electron microscope analyses, and (**C**) distribution of exosomes secreted by ADSCs and measurement of particle diameter by nanoparticle tracking analysis.

Western blotting revealed the expression of the positive exosomal surface markers, including CD9, CD81, flotillin-1, and TSG101 in the isolated exosomes than the control sample as cell lysates, and the expression of the negative control protein calnexin was not observed (Figure 1A). Transition electron microscopy (TEM) showed a round exosome with lipid bilayers and acceptable quality in terms of morphology and size range (Figure 1B). The NTA measurements of exosome size distribution revealed a single peak, with an average size of 90.8 nm ± 38.3 nm (Figure 1C). The quality of the isolated exosomes was good, with a relatively uniform size distribution.

### 2.2. Autophagy Activation in the DRG and Spinal Segments

**Figure 2:**
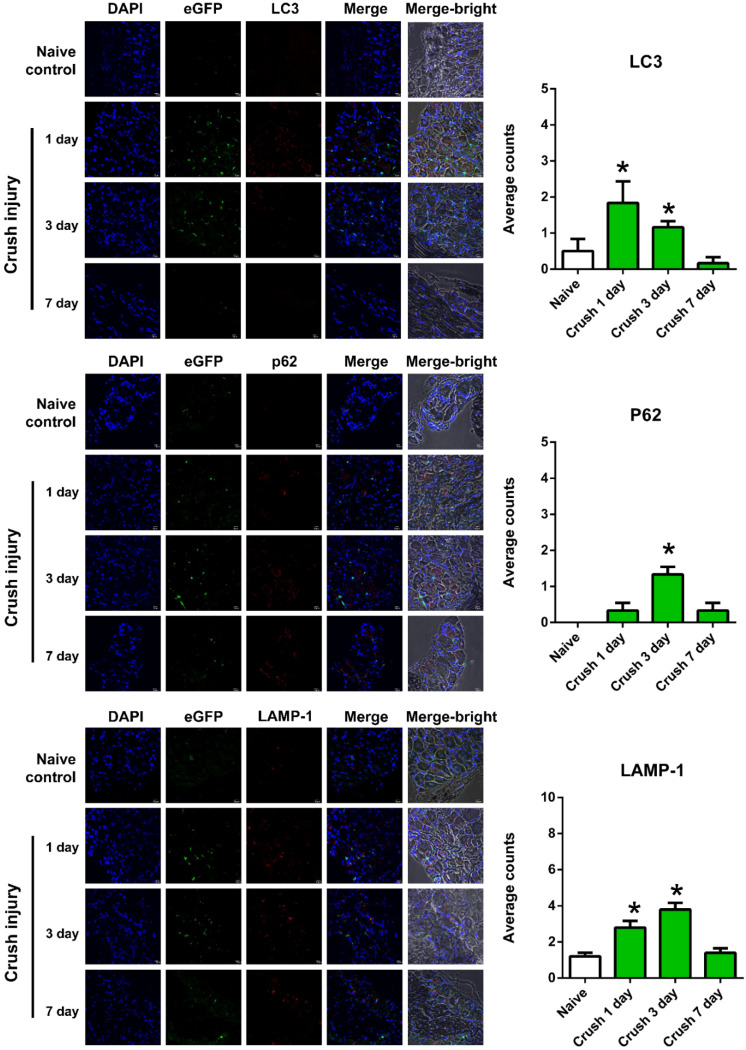
Immnuohistochemistry depicting the co-localization of DAPI and eGFP with autophagy-related markers (LC3, p62, or LAMP-1) in the dorsal root ganglia of L4–6 in the MaFIA mice without nerve crush injury (naïve control) and at 1 d, 3 d, and 7 d following sciatic nerve crush injury. * Indicates a significant change (*p* < 0.05) when compared to those in the naïve control (*n* = 6). The error bar represents the standard error of mean.

According to immunohistomorphometric analysis of the DRG (*n* = 6 for each group of mice), the nerve crush injuries have significantly induced autophagy in macrophages following the nerve crush injury when compared with those in the naive control (Figure 2). Quantification of the number of cells with co-localization of DAPI and eGFP with those autophagy-related markers showed the following patter. Compared with the naive control, the average number of LC3-positive or LAMP-1-positive autophagic cells per field was significantly induced 1 d and 3 d after the crush injury. The average number of p62-positive autophagic cells per field was significantly induced 3 d after crush injury. The number of LC3-, p62-, or LAMP-1-positive autophagic cells was significantly decreased 7 d after the crush injury.

**Figure 3:**
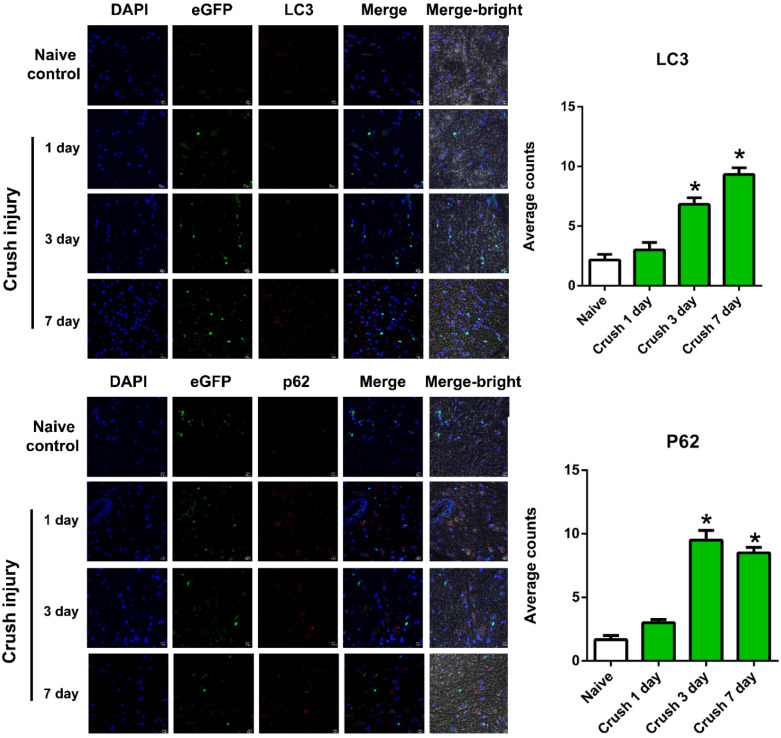

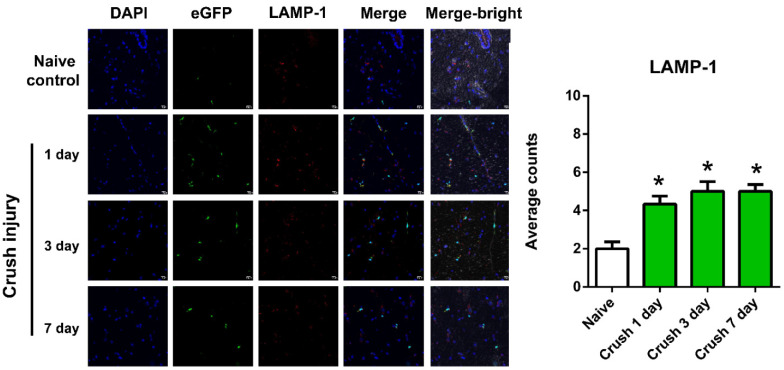
Immunohistochemistry of co-localization of DAPI and eGFP with the autophagy-related markers (LC3, p62, or LAMP-1) in the spinal segment of L4–6 in the MaFIA mice without nerve crush injury (naïve control) and at 1 d, 3 d, and 7 d following sciatic nerve crush injury. * Indicates a significant change (*p* < 0.05) when compared to those in the naïve control (*n* = 6). The error bar represents the standard error of mean.

For the spinal segments (*n* = 6 for each group of mice), nerve crush injuries significantly induced autophagy in macrophages following the nerve crush injury when compared with those in the naive control (Figure 3). Quantification of the number of cells with co-localization of DAPI and eGFP with those autophagy-related markers indicates that, compared to the naive control, the average number of LC3-positive and p62-positive autophagic cells per field was significantly increased at 3 d and persisted at 7 d after the crush injury. The average number of LAMP-1-positive autophagic cells per field was significantly increased at 1 d and persisted at 3 d and 7 d after the crush injury.

### 2.3. ADSC-F-Exo Reduced Autophagy in the Spinal Segments

**Figure 4:**
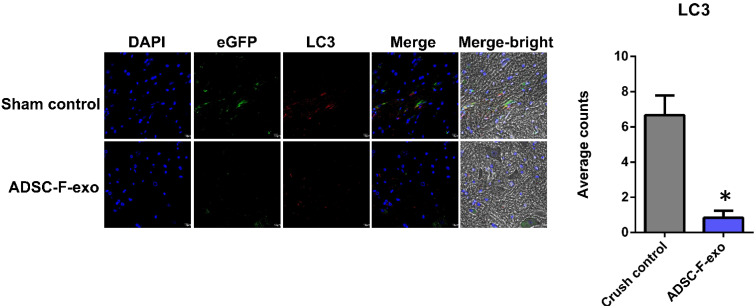

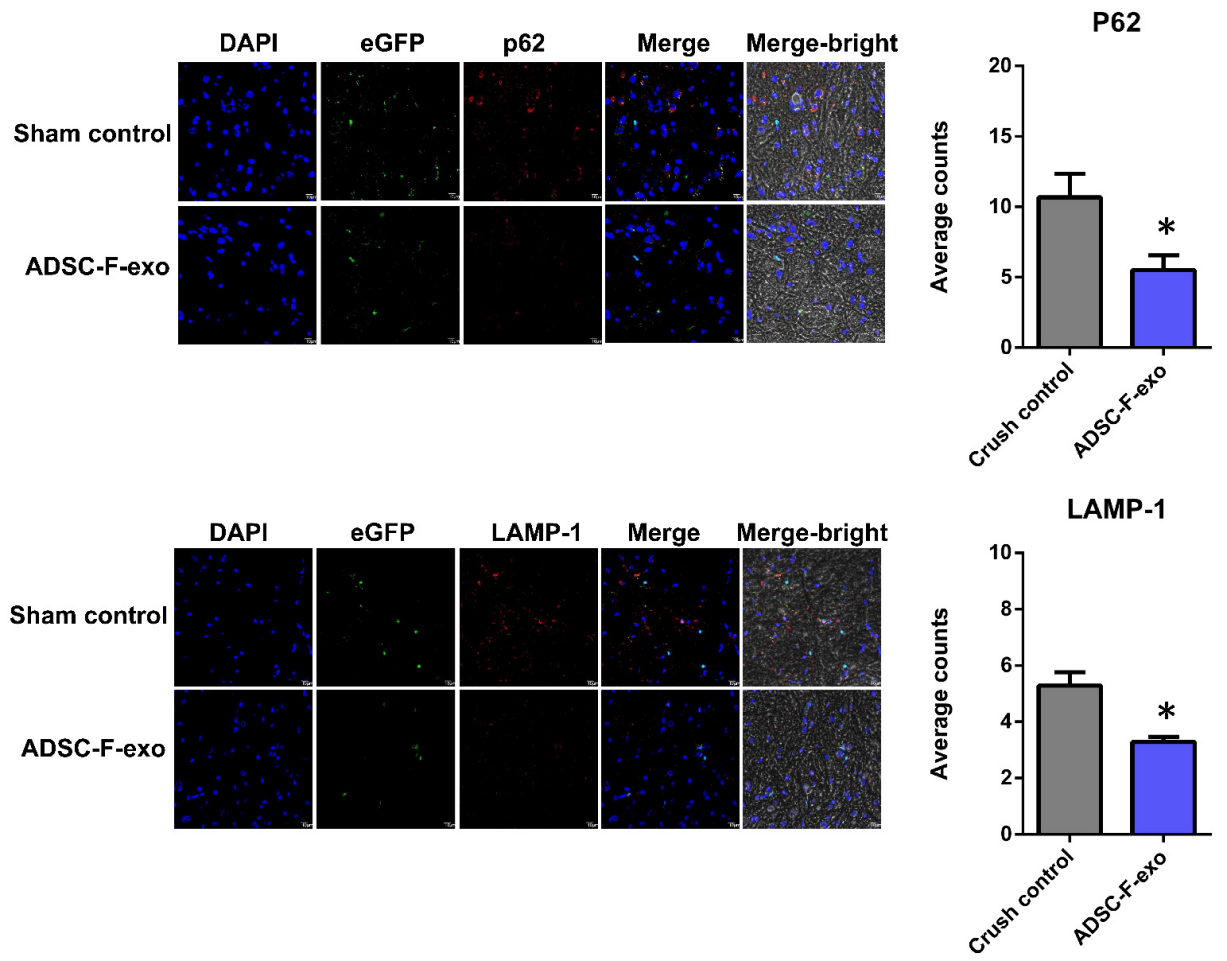
Immunohistochemistry depicting the co-localization of DAPI, eGFP, and the autophagy-related markers (LC3, p62, or LAMP-1) in the spinal segment of L4–6 of MaFIA mice in the absence (crush control) and presence of ADSC-F-exo treatment. * Indicates a significant change (*p* < 0.05) when compared to those in the crush control (*n* = 6). The error bar represents the standard error of mean.

As shown in Figure 4, immunohistomorphometric analysis (*n* = 6 for each group of mice) revealed that the treatment of nerve crush injuries with ADSC-F-exo significantly reduced the degree of macrophage autophagy 3 d following the nerve crush injury, compared with that observed in the crush control mice. Quantification of the number of cells exhibiting the co-localization of DAPI and eGFP with the autophagy-related markers indicates that, compared to the nerve crush control, the average number of autophagic cells per field was reduced after ADSC-F-exo treatment (LC3, 6. 7 ± 1.1 vs. 0.8 ± 0.4, *p* < 0.001; p62, 10.7 ± 1. 7 vs. 5.5 ± 1.1, *p* = 0.026; LAMP-1, 5.3 ± 0.8 vs. 3.3 ± 0.2, *p* = 0.002).

### 2.4. Exosomal Protein Content

**Figure 5:**
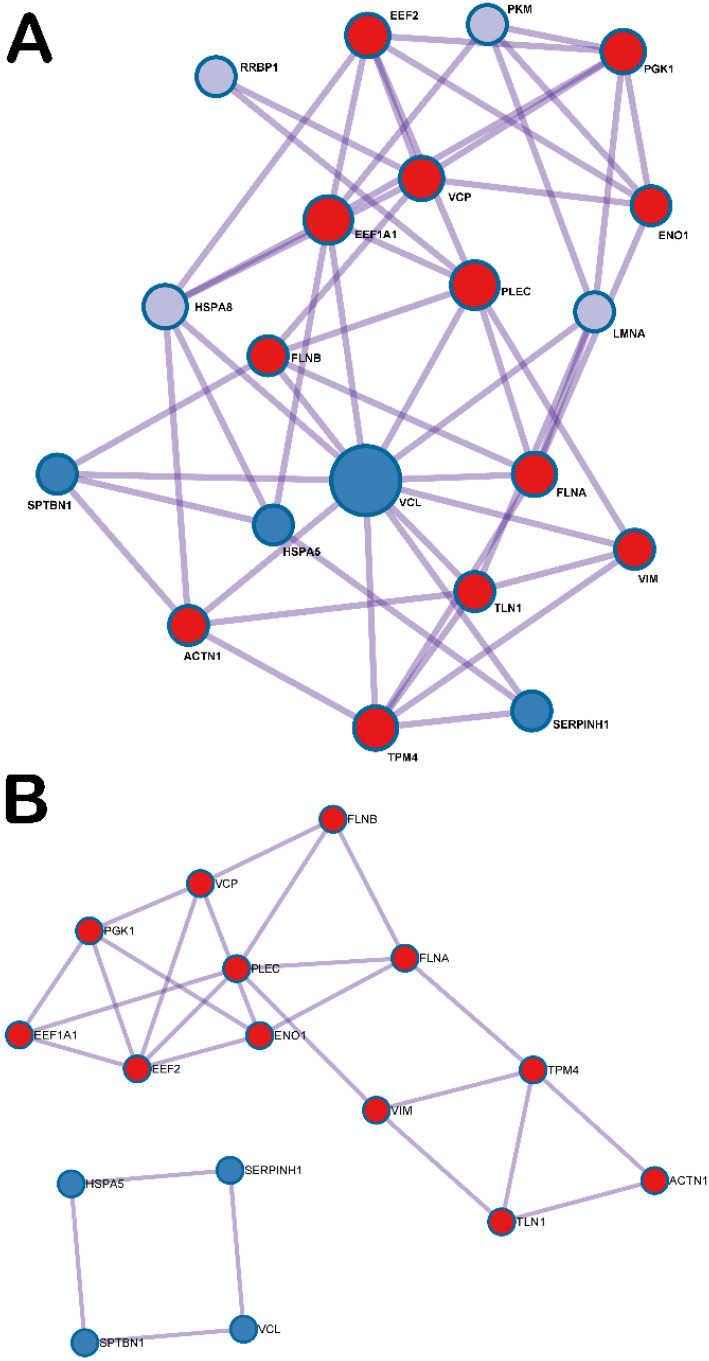
(**A**) Protein-protein interaction network and MCODE components identified from the abundant exosomal protein lists; (**B**) The functional network of the protein-protein interaction.

An iTRAQ-based quantitative proteomic analysis was applied to analyze the expression of exosomal proteins in the ADSC-F-exo samples (*n* = 4). Of the 1306 identified proteins, there were 22 abundant exosomal proteins that had at least 1.5-fold upregulation of cytoplasmic actin protein in all samples (Table 1). Gene annotation and enrichment analysis using Metascape showed that the upregulated genes were considerably enriched in the top 15 pathways (Table 2), with the top five pathways involved in protein methylation, diseases associated with growth factor receptors- and second messengers-mediated signaling, supramolecular fiber organization, platelet degranulation, and glycolysis in senescence. Only one activated transcription factor, i.e., hypoxia-inducible factor 1 alpha (HIF1-α) was detected by transcriptional regulatory relationships unraveled by sentence-based text-mining (TRRUST, http://www.grnpedia.org/trrust, accessed on 30 May 2021) [42] to be involved in these identified proteins. According to the biological function of GO terms, two exosomal proteins, including heat shock protein family A member 8 (HSPA8, GO:1904764 chaperone-mediated autophagy translocation complex disassembly) and eukaryotic translation elongation factor 1 alpha 1 (EEF1A1, GO:1904714 regulation of chaperone-mediated autophagy; GO:0061684 chaperone-mediated autophagy) (Supplemental File S1), are involved in autophagy pathways. The PPI network and MCODE components indicate that the exosomal proteins mainly involved two networks for its functions, whereas one network included PGK1, EEF1A1, EEF2, VCP, PLEC, ENO1, FLNB, FLNA, VIM, TPM4, TLN1, and ACTN1. Meanwhile, the other network included HSPA5, SERPINH1, VCL, and SPTBN1 (Figure 5).

## 3. Discussion

Overall, our findings indicate that nerve crush injuries significantly induce autophagy in the dorsal root ganglia and dorsal horn of the spinal segments. Furthermore, topically sprayed ADSC-F-exo at the crush site significantly reduces the extent of macrophage autophagy in the spine following nerve crush injury. Proteomic analysis of the content of ADSC-F-exo reveals that the transcription factor HIF1-α, along with the abundant exosomal proteins HSPA8 and EEF1A1, may be involved in mediating the autophagy pathway.

The effects of HIF-1α are associated with the activation of autophagy [43]. Previous studies have shown that HIF-1α leads to the transcription of BCL2 interacting protein 3 (BNIP3), which competes with Bcl-2 and Bcl-XL for interaction with Beclin to induce autophagy [43]. HIF-1α-induced autophagy plays an important role in eliminating damaged mitochondria and recruiting normal mitochondria [44]. Cells with a lack of HIF expression exhibit a weakened autophagic response under hypoxic conditions [45]. Importantly, HIF-1α is a critical transcriptional regulator in regenerating neurons [46]. The expression of HIF-1α is increased to exhibit a protective effect after a traumatic spinal cord injury [44] and a traumatic brain injury [47,48,49]. Following nerve axotomy and compression, ATG5 or NAD+-dependent deacetylase sirtuin-1 (SIRT1) overexpression in spinal motoneurons stimulates mTOR-independent autophagy to improve motor axonal regeneration [50].

HSPA8 protein (or 70-kDa heat shock cognate, hsc70) is a constitutively expressed protein that belongs to the heat shock protein 70 (hsp70) chaperone family [51]. HSPA8 is constitutively expressed at high levels in neuronal cell bodies and is enriched in the mammalian nervous system compared to non-neural tissues [52]. It has been reported that HSPA8 acted as an intrinsic protector of neural precursor cells and neuroepithelial cells [53]. It also preserves synaptic function during stress [52], and plays an important role in combating neurodegenerative diseases [54,55]. HSPA8 interacts with Tau, an intrinsically disordered protein that is involved in the stabilization of the axonal microtubules in an aggregated form and drives its clearance by the chaperone-mediated autophagy pathway [55]. An exogenous supply of HSPA8 can reduce the subsequent loss of neurons by apoptosis following a nerve injury [56] and protect the motoneurons from stress [57].

As one of the most abundant translational factors, EEF1A is a GTP-binding protein that is responsible for the delivery of aminoacylated tRNAs to the ribosome to increase the size of nascent polypeptide chains [58]. EEF1A1 is also known to directly bind both pre-existing and newly synthesized defective polypeptides released from ribosomes to generate a signal that induces aggresome formation [59,60], which is initiated upon proteasome failure, and facilitates autophagic clearance of protein aggregates to protect cells from proteotoxicity [59]. EEF1A1 plays a critical role in maintaining long-term synaptic plasticity. Dysregulation of EEF1A1 is involved in the molecular mechanisms behind neurodegenerative diseases, which feature the presence of misfolded polypeptide-containing intracellular inclusion bodies [61,62]. The decreased protein level of EEF1A1 distinguishes autophagy from cell senescence [63]. Moreover, it has been proven that exogenous EEF1A1 expression inhibits caspase-independent cell death [64].

In this study, the PPI network and MCODE components of the 22 identified abundant exosomal proteins of ADSC-F-exo disclosed that mainly two networks were involved in the functions of exosomes, including PGK1, EEF1A1, EEF2, VCP, PLEC, ENO1, FLNB, FLNA, VIM, TPM4, TLN1, and ACTN1; the other network included HSPA5, SERPINH1, VCL, and SPTBN1. Notably, HSPA8 was not included in either network. It is, therefore, reasonable to suggest that the ADSC-F-exo would exhibit some currently under-explored functions other than the autophagy pathways in the treatment of a nerve crush injury. As not all functions of the ADSC-F-exo could be detected in this study, an important limitation should be noted. From this perspective, the exosomal contents may synergistically affect the target cells [65].

The results of this study provide evidence supporting that the exosomes secreted by ADSCs following FK506 treatment reduced autophagy in the spinal segments after nerve crush injury, thus shedding light on the new potential therapeutic application in the regulation of inflammation and neuropathic pain after nerve crush injury. However, before the clinical trial, more studies should be performed to elucidate the mechanism behind. Other limitations of this study should also be acknowledged. First, the autophagy is upregulated to limit the effects of homeostasis perturbation and often plays a protective function in response to cell injury [66]. Despite this, the induction of autophagy may not only generate a hostile microenvironment [67] but also be beneficial in coping with the stress in some neurodegenerative diseases [68]. The role of induced autophagy in macrophages in the milieu surrounding the innervated neurons during peripheral nerve injury remains unexplored in great detail. Second, this study did not discriminate the occurrence of autophagy in a resident or invaded macrophages following a nerve crush injury. As these two types of macrophages have distinct functions in the spine, the detailed information on autophagy for these two types of macrophages has to be further explored. Third, in this study, the autophagy of macrophages was mainly assessed by immunohistochemistry study of the tissue section. However, further studies using mice lacking the specific ligand or receptor of the autophagy pathway may help in providing a more solid conclusion. Fourth, the effect of exosomes secreted from the ADSCs following FK506-induced autophagy may rely on factors other than the protein cargo inside exosomes, such as microRNAs [69] or long noncoding RNAs [70] and should be considered accordingly. Finally, one can expect that the eGFP in the MaFIA mice can be expressed not only in macrophages but also in a small proportion of mice dendritic cells [71]. In turn, it can result in biased outcome measurements. Due to this, a deep exploration of the mechanisms underlying the functions of exosomes secreted by ADSCs under various milieu remains unexplored and urgent.

## 4. Materials and Methods

### 4.1. Cultured Mouse ADSCs

ADSCs were purchased from iXCells Biotechnologies (MADSC-bf, San Diego, CA, USA). The ADSCs were isolated from the interscapular brown fat tissues of C57BL/6 mice. At the beginning of the experiments, 1 × 10^4^ ADSCs were expanded for subsequent passages using ADSC basal medium (Cat # MD-0003) according to the manufacturer’s instructions provided by iXcells Biotechnologies. The cells previously tested positive for stem cell markers CD105, CD73, CD90, and CD44, and negative for CD3, CD11b, CD25, CD45, and CD106 by flow cytometric analysis.

### 4.2. Exosome Isolation

The exosomes (defined as ADSC-F-exo) secreted by ADSCs following treatment with 100 µg/mL FK506 (InvivoGen, Hong Kong, China) in dimethyl sulfoxide (DMSO) for 24 h were isolated using ExoQuick-TC^TM^ exosome precipitation solution (EXOTC50A-1, System Biosciences), according to the manufacturer’s instructions. With the concentration of 1 × 10^9^ to 1 × 10^10^ exosomes in 100 uL of the culture media, the media were centrifuged at 3000× *g* for 15 min, and the supernatant was transferred into a new tube, followed by the addition of equal volumes of the ExoQuick-TC^TM^ solution. After mixing, supernatants were refrigerated at 4 °C overnight for at least 12 h and then centrifuged at 1500× *g* for 30 min. The supernatant was discarded, and the pellet was resuspended in PBS and used for further experiments.

### 4.3. Characterization of Exosomes

Characterization of isolated exosomes was based on the Guidelines of the Minimal Information for Studies of Extracellular Vesicles (MISEV2018) [72]. Expression of exosomal surface markers on isolated 30 ug exosomes was detected by Western blotting in triplicate, with the culture medium used as a control. For exosomes, total protein was separated by polyacrylamide gel electrophoresis and electrotransferred to polyvinylidene fluoride (PVDF) membranes (Millipore, Billerica, MA, USA). The membranes were blocked with 5% skim milk in PBS/Tween-20 and incubated with primary antibodies against CD9 (cat # ab92726, 1:1000; Abcam, Cambridge, MA, USA), CD81 (cat # ab109201, 1:1000; Abcam), Flotillin-1 (cat # 18634, 1:1000; Cell Signaling Technology, Danvers, MA, USA), TSG101 (cat # ab30871, 1:1000; Abcam), and the negative control protein calnexin (cat # ab22595, 1:1000; Abcam) at 4 °C overnight. Then, membranes were washed with 0.1% TBS/Tween 20 for 10 min, three times at room temperature, and incubated with horseradish peroxidase (HRP)-conjugated secondary antibodies (cat # NA931; GE Healthcare Amersham, Piscataway, NJ, USA) for 2 h at 37 °C, and quantified using a FluorChem SP imaging system (Alpha Innotech, San Leandro, CA, USA).

For TEM analyses, exosomes (10 µL) were fixed with 2.5% glutaraldehyde for 2 h and added to a 200 mesh Formvar stabilized with carbon. The grids were stained with 2% uranyl acetate for 1 h. The samples were analyzed using a transmission electron microscope (HT-7700; Hitachi, Tokyo, Japan) at 100 kV.

The size and concentration of exosomes were analyzed using a Malvern NanoSight NS300 nano tracking analyzer (NanoSight, Amesbury, UK), and the samples were injected into the sample chamber with sterile syringes (BD Discardit II, Franklin Lakes, NJ, USA) until the liquid reached the tip of the nozzle. The size distribution and concentration of exosomes in the liquid suspension were measured according to the properties of both light scattering and Brownian motion. All measurements of particle movement were detected by a 488 nm laser at 20–100 particles/frame and 30 frames per second for 1 min at room temperature. The software used for capturing and analyzing the data was NTA 3.1 Build 3.1.54.

### 4.4. Animal Nerve Crush Surgery

*CSF-1R–GFP*^+^ macrophage Fas-induced apoptosis (MaFIA) transgenic mice were purchased from The Jackson Laboratory (stock #005070, JAX, Bar Harbor, ME, USA). The MaFIA transgenic mouse model was developed by placing genes encoding enhanced eGFP and mutant human FK506-binding protein (FKBP)–Fas suicide construct under the macrophage-specific mouse colony stimulating factor 1 receptor promoter (Csf1r) [73]. Macrophages expressing eGFP can be efficiently detected by fluorescence microscopy [71]. The nerve crush injury model was established in 8–12-week-old male mice, weighing between 20 and 30 g, and performed as in our previous reports [74,75]. Anesthesia was induced by intramuscular injection of 25 mg/kg ketamine and 50 mg/kg xylazine. Then, the right sciatic nerve of the mouse was exposed at the mid-thigh level and crushed with No. 5 Jeweler forceps, using consistent pressure for 30 s.

The DRG and spinal segments of L4–6 of MaFIA transgenic mice receiving the right sciatic nerve were harvested at 1 d, 3 d, and 7 d (*n* = 6 for each group of mice) for immunohistochemical analysis to detect autophagy in macrophages. DRG and spinal segments from additional MaFIA transgenic mice that received surgery were harvested as naïve controls (*n* = 6).

For the ADSC-F-exo treatment groups, exosomes (100 µg) resuspended in 100 µL PBS were sprayed around the crushed nerve segment using a 30-gauge syringe needle (Becton-Dickinson & Co, Franklin Lakes, NJ, USA). Mice with crushed nerve segments sprayed with 100 µL PBS were used as the crush control. Three days after the crush injury (the day was decided according to the expression data in the DRG and spinal segments from the MaFIA mouse model with nerve crush injury), the spinal segment of L4–6 from the right side of mice of the ADSC-F-exo treatment groups and crush control group were dissected. The harvested spinal segments were used for immunohistochemistry and for the detection of neurotrophins. All housing conditions, surgical procedures, analgesia, and assessments were performed in an AAALAC-accredited specific pathogen-free facility, following national and institutional guidelines. The animal protocols were approved by the IACUC of Kaohsiung Chang Gung Memorial Hospital, Taiwan.

### 4.5. Immunohistochemistry of Autophagy-Related Markers in Spinal Segment

For immunohistochemistry, sections of DRG and spinal segment of L4–6 at 8 micrometer thickness were used. The frozen sections were washed with PBS-Tween 20. The slices were blocked with PBS containing 1% Triton X-100 and 5% bovine serum albumin. The cells were, then, incubated with the indicated primary antibodies, including LC3A/B (Abcam-ab128025), p62 (Abcam-ab91526), and LAMP-1 (Abcam-ab25245), in a moisture chamber at 4 °C overnight. After washing, the sections were incubated with a secondary antibody (Biolegend, San Diego, CA, USA) for 1 h at room temperature. In combination with the staining with 4′,6-diamidino-2-phenylindole (DAPI mounting medium, VECTOR-H1200), we analyzed the localization of these autophagy-related markers in cells expressing eGFP using a confocal microscope (FLUOVIEW FV10i, Olympus, Tokyo, Japan). Quantification of the number of cells that showed co-localization of DAPI, eGFP, and the autophagy-related markers (LC3, p62, or LAMP-1) was performed based on 20 randomly selected fields of the DRG or dorsal horn of spinal segments at 60× magnification and expressed as an average number of cells per field.

### 4.6. Extraction of Exosomal Protein and iTRAQ Labeling

Exosomal proteins of ADSC-F-exo were purified using T-PER Tissue Protein Extraction Reagent (78510, Thermo Fisher Scientific, Waltham, MA, USA). Protein samples were desalted using Amicon^®^ Ultra-15 (Millipore, Burlington, MA, USA) and quantified using the BCA protein assay (23,225, Thermo Fisher Scientific, Waltham, MA, USA). For iTRAQ labeling, 25 µg of the protein samples were dried using SpeedVac and resuspended in the iTRAQ dissolution buffer, which included 0.5 M triethylammonium bicarbonate (TEAB; pH 8.5). Protein samples were reduced using the iTRAQ reduction buffer (tris-2-carboxyethyl phosphine, TCEP) at 60 °C for 30 min and then alkylated in the dark using iodoacetamide at 37 °C for 30 min. After protein digestion using sequencing-grade modified trypsin (V511A, Promega, Madison, WI, USA), the samples were dried using SpeedVac. Next, the peptides were reconstituted in the iTRAQ dissolution buffer and labeled using iTRAQ labeling reagents, according to the manufacturer’s instructions (Applied Biosystems Inc., Foster City, CA, USA).

### 4.7. Two-Dimensional Liquid Chromatography with Tandem Mass Spectrometry (2D LC-MS/MS)

The iTRAQ-labeled samples were analyzed using a Q Exactive^TM^ HF mass spectrometer (Thermo Fisher Scientific) coupled with an UltiMate™ 3000 RSLCnano HPLC System (Thermo Fisher Scientific). The iTRAQ-labeled peptides were pooled and desalted using Sep-Pak C18 cartridges (Waters, Milford, MA, USA). The desalted peptides were dried using SpeedVac and resuspended in 0.5% trifluoroacetic acid. The peptide mixtures were loaded onto an EASY-Spray™ C18 column (Thermo Fisher Scientific) and separated using a 0.1% formic acid solution with varying amounts of acetonitrile (5–80%). The top 15 most abundant precursor ions within the 375–1400 *m*/*z* scan range were dynamically selected for further fragmentation in high collision dissociation (HCD) mode, with the normalized collision energy set to 33 ± 1%. In the full MS scan, the resolution was set to 60,000 at *m*/*z* 200, AGC target to 3 × 10^-6^, and maximum injection time to 50 ms. For the MS/MS scan, the resolution was set to 15,000, AGC target to 5 × 10^-4^, and the maximum injection time was set to 100 ms. The release of the dynamic exclusion of selected precursor ions was set to 20 s.

### 4.8. Database Search and Protein Quantification

Raw MS data were examined using the Mascot search algorithm (version 2.5, Matrix Science, Chicago, IL, USA) against the Swiss-Prot human protein database using Proteome Discoverer (version 2.1, Thermo Fisher Scientific) software. For protein identification, the search parameters were set as follows: carbamidomethylation at cysteine as the fixed modification, oxidation at methionine, acetylation at protein N-terminus, iTRAQ-labeled at peptide N-terminus, lysine residue as dynamic modifications, 10 ppm and 0.02 Da for MS/MS tolerance, and maximum missing cleavage sites with two. These exosomal proteins with at least 1.5-fold upregulation of the actin protein inside the exosome in quadruplicate samples were identified as abundant exosomal proteins.

### 4.9. Gene Annotation, Pathway and Process Enrichment Analysis, and Protein-Protein Interaction Enrichment Analysis

Gene annotation and enrichment analysis of the pathway, process, and protein-protein interaction (PPI) were carried out using Metascape (http://metascape.org, accessed on 30 May 2021) [76], an integrated website of a broad set of current biological databases, and the application of a robust analytical pipeline to produce readily interpretable results. In Metascape, the analytical conditions were as follows: minimal overlap = 3, minimal enrichment = 1.5, and threshold of *p* = 0.01. In Metascape, enrichment analysis of PPI was carried out using the following databases: STRING [77], BioGrid [78], OmniPath [79], and InWeb_IM [80], using the Molecular Complex Detection (MCODE) algorithm [81], which is applied to find clusters with highly interconnected regions in a network. In Metascape, the major trans-acting factors in transcriptional regulation of these identified exosomal proteins were detected by TRRUST [42], containing 6552 transcription factor target interactions for 828 mouse transcription factors, were used for identification.

### 4.10. Statistical Analysis

All results are provided as a mean ± standard error. An overall analysis of the differences between group means was performed using one-way analysis of variance (ANOVA), followed by a post hoc Fisher’s least significant difference test. Statistical significance was set at *p* < 0.05.

## 5. Conclusions

This study reported that locally applied ADSC-F-exo significantly reduced autophagy of macrophages in the spinal segments after nerve crush injury. Proteomic analysis of ADSC-exo showed that HSPA8 and EEF1A1 are potential candidates involved in the exosome-mediated reduction in autophagy.

## Figures and Tables

**Table 1:** The abundant exosomal proteins that exhibited at least 1.5-fold upregulation than the cytoplasmic actin protein in the exosomes secreted by ADSCs following in FK506 treatment.

Accession	Description	Gene Name	Unique Peptides	Fold of Abundances
P20152	Vimentin	Vim	36	5.10
Q01853	Transitional endoplasmic reticulum ATPase	Vcp	48	5.69
Q9QXS1	Plectin	Plec	176	4.89
Q7TPR4	Alpha-actinin-1	Actn1	36	3.52
P52480	Pyruvate kinase PKM	Pkm	37	3.83
Q8BTM8	Filamin-A	Flna	95	3.75
P16546	Spectrin alpha chain, non-erythrocytic 1	Sptan1	111	3.19
Q64727	Vinculin	Vcl	59	2.41
Q62261	Spectrin beta chain, non-erythrocytic 1	Sptbn1	82	2.97
P20029	78 kDa glucose-regulated protein	Hspa5	35	3.38
P26039	Talin-1	Tln1	90	2.58
P17182	Alpha-enolase	Eno1	16	2.45
Q80X90	Filamin-B	Flnb	92	2.26
P63017	Heat shock cognate 71 kDa protein	Hspa8	26	2.01
P10126	Elongation factor 1-alpha 1	Eef1a1	20	2.22
Q99PL5	Ribosome-binding protein 1	Rrbp1	63	2.13
Q6IRU2	Tropomyosin alpha-4 chain	Tpm4	16	2.07
P58252	Elongation factor 2	Eef2	44	1.93
P48678	Prelamin-A/C	Lmna	34	2.00
P15331	Peripherin	Prph	4	1.57
P09411	Phosphoglycerate kinase 1	Pgk1	24	1.87
P19324	Serpin H1	Serpinh1	25	1.67
P60710	Actin, cytoplasmic 1	Actb	9	1.00

**Table 2:** Top 15 clusters with their representative enriched terms (one per cluster).

GO	Description	Count	%	Log10(*p*)	Log10(*q*)
R-HSA-8876725	Protein methylation	4	20	−9.15	−4.79
R-HSA-5663202	Diseases of signal transduction by growth factor receptors and second messengers	7	35	−7.90	−3.84
GO:0097435	Supramolecular fiber organization	8	40	−7.65	−3.82
R-HSA-114608	Platelet degranulation	5	25	−7.56	−3.82
WP5049	Glycolysis in senescence	3	15	−7.30	−3.81
GO:0002064	Epithelial cell development	5	25	−6.36	−3.26
R-HSA-111465	Apoptotic cleavage of cellular proteins	3	15	−5.59	−2.74
R-HSA-445355	Smooth Muscle Contraction	3	15	−5.53	−2.68
GO:0051129	Negative regulation of cellular component organization	6	30	−5.01	−2.37
GO:0003012	Muscle system process	5	25	−4.88	−2.29
GO:0045727	Positive regulation of translation	3	15	−3.93	−1.55
GO:0031667	Response to nutrient levels	4	20	−3.52	−1.23
GO:1903827	Regulation of cellular protein localization	4	20	−3.33	−1.07
GO:0071417	Cellular response to organonitrogen compound	4	20	−3.03	−0.79
R-HSA-1474244	Extracellular matrix organization	3	15	−2.92	−0.71

“Count” is the number of genes in the identified protein lists with membership in the given ontology term. “%” is the percentage of all of the identified protein that are found in the given ontology term (only input genes with at least one ontology term annotation are included in the calculation). “Log10(*p*)” is the *p*-value in log base 10. “Log10(*q*)” is the multi-test adjusted *p*-value in log base 10.

## Data Availability

The data presented in this study are available on request from the corresponding author.

## Supplementary Materials

The following are available online at https://www.mdpi.com/article/10.3390/ijms22179628/s1.
